# Therapeutic Nanoparticles and Their Targeted Delivery Applications

**DOI:** 10.3390/molecules25092193

**Published:** 2020-05-08

**Authors:** Abuzer Alp Yetisgin, Sibel Cetinel, Merve Zuvin, Ali Kosar, Ozlem Kutlu

**Affiliations:** 1 Materials Science and Nano-Engineering Program, Faculty of Engineering and Natural Sciences, Sabanci University, Istanbul 34956, Turkey; yalp@sabanciuniv.edu; 2 Nanotechnology Research and Application Center (SUNUM), Sabanci University, Istanbul 34956, Turkey; cetinel@sabanciuniv.edu; 3 Mechatronics Engineering Program, Faculty of Engineering and Natural Sciences, Sabanci University, Istanbul 34956, Turkey; mervezuvin@sabanciuniv.edu (M.Z.); ali.kosar@sabanciuniv.edu (A.K.); 4 Center of Excellence for Functional Surfaces and Interfaces for Nano Diagnostics (EFSUN), Sabanci University, Istanbul 34956, Turkey

**Keywords:** nanotechnology, therapeutic nanoparticles, targeted delivery, diseases treatment

## Abstract

Nanotechnology offers many advantages in various fields of science. In this regard, nanoparticles are the essential building blocks of nanotechnology. Recent advances in nanotechnology have proven that nanoparticles acquire a great potential in medical applications. Formation of stable interactions with ligands, variability in size and shape, high carrier capacity, and convenience of binding of both hydrophilic and hydrophobic substances make nanoparticles favorable platforms for the target-specific and controlled delivery of micro- and macromolecules in disease therapy. Nanoparticles combined with the therapeutic agents overcome problems associated with conventional therapy; however, some issues like side effects and toxicity are still debated and should be well concerned before their utilization in biological systems. It is therefore important to understand the specific properties of therapeutic nanoparticles and their delivery strategies. Here, we provide an overview on the unique features of nanoparticles in the biological systems. We emphasize on the type of clinically used nanoparticles and their specificity for therapeutic applications, as well as on their current delivery strategies for specific diseases such as cancer, infectious, autoimmune, cardiovascular, neurodegenerative, ocular, and pulmonary diseases. Understanding of the characteristics of nanoparticles and their interactions with the biological environment will enable us to establish novel strategies for the treatment, prevention, and diagnosis in many diseases, particularly untreatable ones.

## 1. Introduction

Nanomedicine is an emerging approach for the implementation of nanotechnological systems in disease diagnosis and therapy. This branch of nanotechnology can be classified in two main categories: nanodevices and nanomaterials. Nanodevices are miniature devices at nanoscale including microarrays [1,2] and some intelligent machines like respirocytes [3]. Nanomaterials contain particles smaller than 100 nanometers (nm) in at least one dimension.

Recent explorations of biomedical science resulted in the successful improvement of therapeutic agents’ design in disease treatment. However, there is a major obstacle before the treatment efficiency of various diseases, which is the delivery of therapeutic agents to the target area. The application of conventional therapeutic agents has limitations such as non-selectivity, undesirable side effects, low efficiency, and poor biodistribution [4]. Therefore, the focus of current research activities is to design well-controlled and multifunctional delivery systems. Association of therapeutic agents with nanoparticles exhibiting unique physicochemical and biological properties and designing their pathways for suitable targeting is a promising approach in delivering a wide range of molecules to certain locations in the body [5]. This targeted strategy enhances the concentration of therapeutic agent in cells/tissues; thereby, low doses can be used, particularly if there is a contradiction between the therapeutic activity and the toxic effects of the agent. Increasing concentration of therapeutic agents in-target location also improves their therapeutic index by enhancing the efficacy and/or increasing the tolerability in biological systems. Water-insoluble therapeutic agents can also be combined with nanoparticles, which can protect them from physiological barriers and improve their bioavailability. On the other hand, association of therapeutic nanoparticles with contrast agents provides a way of tracking their pathway and imaging their delivery location in in vivo systems.

The aforementioned advantages enable the targeted therapeutic nanoparticles utilization in various fields of medicine. Here, we summarized the physicochemical properties of nanoparticles making them crucial vehicles in nanomedicine and provide a review of the last decade for therapeutic nanoparticles and their targeted delivery applications in various disorders such as cancer and neurodegenerative diseases.

## 2. Designing Nanoparticles for Therapeutics

Targeted therapy in disease treatment is the approach of delivering appropriate amounts of therapeutic agent for a prolonged period to the affected area within the body. To achieve this, development of safer and more effective therapeutic nanoparticles is crucial and one of the ultimate goals of nanomedicine.

As soon as nanoparticles enter to the bloodstream, they are prone to aggregation and protein opsonization (protein binding to nanoparticle surface as a tag for immune system recognition). The opsonized nanoparticles could be cleared from the bloodstream by phagocytosis or filtration in the liver, spleen, and kidney. This rapid and non-specific clearance by the immune system results in decreased retention time and thus limits bioavailability. By decorating the nanoparticle surface with polyethylene glycol (PEG), carbohydrates, acetyl groups, or protein moieties (arginine-glycine-aspartate (RGD) peptide, albumin), retention time can be altered [6]. However, such surface modification can also alter the recognition ability for targeted delivery. Thus, the cleanability and biodistribution of therapeutic nanoparticles should be well concerned during the design process.

Size is another important factor playing role in controlling circulation and biodistribution of therapeutic nanoparticles. Nanoparticles smaller than 10 nm, can be easily cleared by physiological systems (filtration through the kidney), while particles larger than 200 nm may be cleared by phagocytic cells in the reticuloendothelial system (RES). Accordingly, therapeutic nanoparticles with a size of ˂100 nm have longer circulation time in the bloodstream. Many studies reported that therapeutic nanoparticles in 20–200 nm size showed a higher accumulation rate in tumors because they cannot be recognized by the RES and filtrated by the kidney [7,8,9]. Additionally, blood vessels in tumor areas are higher in number and larger in volume compared to normal tissues. Therefore, nanoparticles with appropriate sizes can reach to the tumor area relatively easily and accumulate for a longer time, which is known as the enhanced permeability and retention (EPR) effect [10,11]. In fact, passive targeting is utilized to accumulate nanoparticles to the tumor site, which is done without functionalizing nanoparticles with targeting moiety. However, in active targeting, the surface of the nanoparticle is coupled with at least one kind of targeting moiety such as, proteins, peptides, nucleic acids, antibodies, or small molecules [12].

Most of the nanoparticles are taken up within the cells by endocytosis through either clathrin- or caveolae-dependent mechanisms [13]. The shape of nanoparticles is also critical for biodistribution due to their internalization by the targeted cells. For instance, rod-shaped cationic nanoparticles are easier targets for endosomal uptake than cationic nanoparticles of other shapes, suggesting that these nanoparticles may be comprehended by immune system cells as rod-shaped bacteria [14].

Surface charge of therapeutic nanoparticles plays an important role in their clearance and targeted delivery. Positively charged nanoparticles generate a higher immune response compared to neutral or negatively charged nanoparticles. Additionally, nanoparticles with a surface potential between −10 and +10 mV are shown to be less susceptible to phagocytosis and non-specific interactions [7,9]. However, the ideal range could depend on the nanoparticle material. Surface charge is also closely related to pH sensibility of nanoparticles. Such nanoparticles can be designed to recognize and locate in specific compartments of the cell. For example, acidic nanoparticles can be targeted to endosomes or lysosomes for releasing their cargo, each of which has a pH of <6.0 [8,15,16].

Surface modification of nanoparticles with long-chain polymers such as polyethylene glycol (PEG) was shown to minimize non-specific protein absorption onto the nanoparticle surface. Due to its intrinsic physicochemical properties, PEG is a favorable polymer for therapeutic nanoparticles, which decreases their phagocytic uptake and reduces their accumulation in non-target organs [17]. Factors such as length, shape, and density of PEG chains affecting surface hydrophilicity and phagocytosis, should be considered before PEGylation of therapeutic nanoparticles. Conjugation of targeting ligands to the surface of PEGylated nanoparticles can improve the target-specific delivery of nanoparticles; however, it also affects their biodistribution.

## 3. Types of Therapeutic Nanoparticles

**Figure 1:**
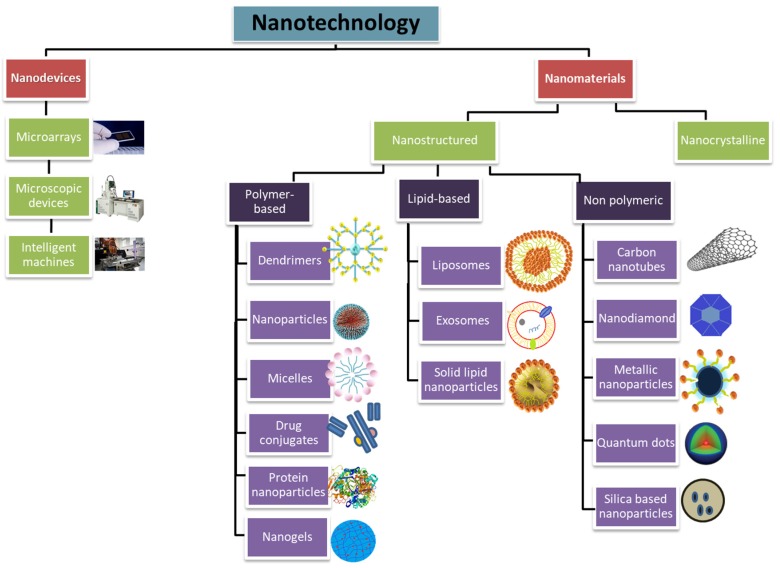
Elements of nanotechnology, which are utilized in therapeutic applications.

**Figure 2:**
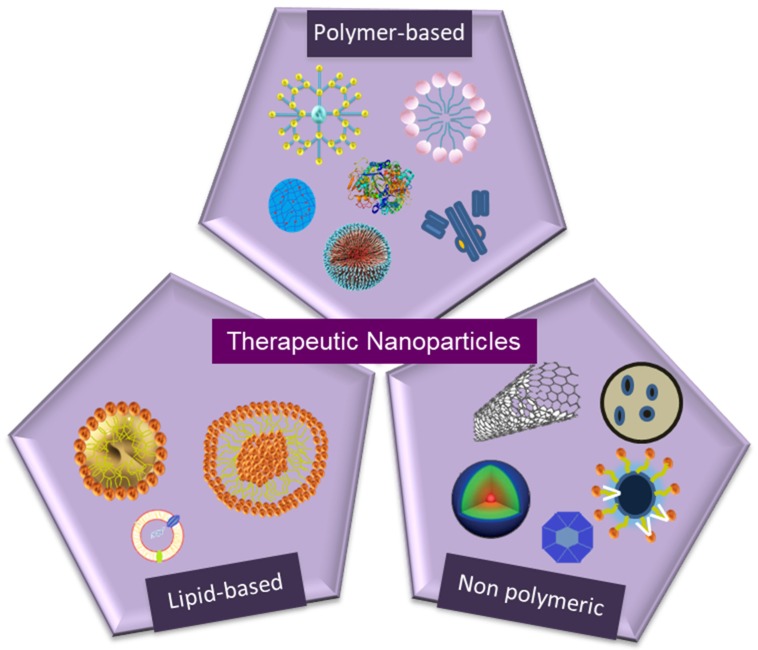
Schematic representations of the therapeutic nanoparticles.

Nanomaterials can be classified in two main categories: Nano-structured and nanocrystalline. Nano-structured materials can further be categorized into polymer-based, non-polymeric, and lipid-based nanoparticles. Polymer-based nanoparticles include dendrimers, nanoparticles, micelles, nanogels, protein nanoparticles, and drug conjugates. Non-polymeric nanoparticles include carbon nanotubes, nanodiamonds, metallic nanoparticles, quantum dots, and silica-based nanoparticles. Lipid-based nanoparticles can be divided into liposomes and solid lipid nanoparticles. So far, the majority of the nanoparticles clinically approved for therapeutic use are polymer-based or lipid-based components. Apart from polymer-based, non-polymeric, or lipid-based nano-structured particles, nanocrystalline particles that are formed by the combination of therapeutic agents in crystalline form are also used in some clinical applications (Figure 1 and Figure 2). In this section, we summarized different types of clinically used nanoparticles and their specificity for therapeutic applications, as well as their current delivery strategies in challenging pathophysiological conditions.

### 3.1. Nano-Structured Particles

#### 3.1.1. Polymer-Based Particles

##### Dendrimers

Dendrimers are widely used polymers in clinical applications due to their hyperbranched, compartmentalized structure, and high monodispersity. Controlling the number of branches in these polymer-based nanoparticles allows their fabrication in very small sizes (1–5 nm). They can be fabricated by polymerization in spherical shape, which leads to the formation of cavities within the dendrimer molecule. Thus, high entrapment efficiency is obtained with high-generation dendrimers, e.g. dendrimers containing more than 64 surface groups, when compared to smaller dendrimers and these are used for the delivery of therapeutic agents. Additionally, dendrimers contain free end groups, which can be easily modified/used for the conjugation of biocompatible compounds to enhance low cytotoxicity and high bio-permeability of the molecule. Such surface modifications can also be applied to improve the target-specific delivery of therapeutic agents. Assembling dendrimers by either encapsulation or complexation makes them attractive vehicles for the concomitant delivery of biologically active molecules such as vaccines, drugs, and genes to the target locations. Currently, mono- or copolymers, such as polyethyleneimine, polyamidoamine, poly(propyleneimine), chitin, etc., are used for therapeutic applications in the form of dendrimers [18,19].

##### Nanoparticles

Polymer-based nanoparticles, synthetic or natural, provide an alternative way for therapeutic applications due to certain characteristics, such as biocompatibility, non-immunogenicity, non-toxicity, and biodegradability [20]. In order to decrease the immunogenicity and toxicity of synthetic polymers, like polycaprolactone (PCL), polylactic acid (PLA), and their monomers, the polyester forms are used. On the other hand, natural polymer-based nanoparticles such as chitosan, gelatin, albumin, and alginate seem to overcome toxicity issues and provide significant improvement in the efficiency of therapeutic agents compared to conventional methods. Polymeric nanoparticles are considered as the matrix system, in which the matrix is uniformly dispersed. They can be classified as nanocapsules or nanospheres depending on their composition. In nanocapsules, a unique polymer membrane encloses therapeutic agents, whereas the therapeutic agents are directly dispersed throughout or within the polymer matrix in nanospheres [21]. Existence of a multitude of preparation methods of polymeric nanoparticles can control the release characteristics of incorporated therapeutic agents, which allows the delivery of a higher concentration of agents to the target location. Moreover, the surface of polymeric nanoparticles could be easily modified and functionalized with a specific recognition ligand which increases the specificity of therapeutic agents in targeted tissue.

##### Micelles

Polymeric micelles are mostly used for the systemic delivery of water-insoluble therapeutic agents. They are in ˂100 nm size and formed in solution as aggregates. The component molecules of polymeric micelles are arranged in spheroidal structure, in which a mantle of hydrophilic groups surrounds hydrophobic cores. The existence of hydrophilic surface contributes to their protection from nonspecific uptake by the reticuloendothelial system ensuring their high stability within physiological systems. On the other hand, the hydrophobic core of polymeric micelles can physically trap the water-insoluble, hydrophobic therapeutic agents. The component molecules can also be covalently linked to this hydrophobic core. Consequently, the dynamic structure of polymeric micelles provides a prominent delivery system for therapeutic agents, which allows versatile loading capacity, conjugation of targeted ligands, and lower rate of dissolution [22].

##### Drug Conjugates

Conjugation of polymers with drug molecules is generally used for low molecular weight agents, particularly in cancer treatment. This conjugation increases the overall molecular weight of drugs, which induces the pharmacokinetic disposition in the cells. Polymer-drug conjugates serve as carriers with high solubility and stability and promote an EPR effect in cancer cells [23]. Covalently conjugated polymer-drugs are shown to be more reliable for sustained drug release and enhanced drug capacity [24]. There are pH-sensitive polymeric drug conjugates, which are made by utilizing pH-responsive chemical bonds between polymer and drug. Thus, the pH sensitivity of the nanoparticle is used for controlled drug release in the tumor site due to its acidic environment [25,26]. It is also reported that polymeric drug conjugates increase the bioavailability of the drug, for instance, shown with paclitaxel and doxorubicin combination therapy [24,27,28].

##### Protein Nanoparticles

Viruses are very efficient and natural carrier systems for transferring their genetic material, which is encapsulated by the capsid proteins. Virus-like particles (VLP), a type of protein nanoparticles, are defined as nano-carrier systems, which have a morphologically similar, virus-isolated structure but do not include the viral genetic material [29,30]. Additionally, caged proteins (CP) are defined as self-assembled protein nanostructures, which are morphologically similar to viruses, however, not deriving from viruses. The VLPs and CPs are attractive nano-carrier systems for the development of the vaccines for cancer because they can induce antigen-specific immune responses against cancer cells [31]. Moreover, there are protein nanoparticles made by self-assembly of protein polymers, which are isolated proteins from animal or plant origin such as collagen, gelatin, silk, albumin, elastin, and soy. Through genetic engineering, protein polymers are self-assembled into functional drug delivery carriers with advantages of polymer-based nanoparticles [32,33]. Abraxane^®^ is an FDA-approved protein nanoparticle drug, enabling paclitaxel delivery by albumin. On the other hand, an HIV vaccine made from VLPs led to critical developments, which accelerated research on protein nanoparticles for clinical use [34].

##### Nanogels

The gels are defined as non-fluid colloidal or polymeric networks that swell when in contact with fluid. A nanogel is considered as a particle of gel with similar properties, however, with a diameter of less than 100 nm by the International Union for Pure and Applied Chemistry (IUPAC) [35]. The swelling property with flexible size and high water content of the nanogels is the result of physically or chemically cross-linked natural or synthetic polymers [36,37]. The first reported nanogel was prepared by physical cross-linking of amphiphilic polysaccharides, where cholesterol-bearing pullulans are self-assembled (by hydrophobic interactions) into nanogels in water [36,38]. The nanogels have some advantages compared to other nano-carrier systems such as decreased untimely drug leakage, encapsulating various therapeutic molecules in the same formulation, and easy administration through parental or mucosal routes. The nanogels are used in various applications, including biosensors, biochemical separation, cell culture, bio-catalysis, drug delivery, antitumor therapy, and so on. Among these, delivery of therapeutics such as nucleic acids, vaccines, cytokines, and nasal vaccines are the most widely studied applications of nanogels [36,37,39].

#### 3.1.2. Non-Polymeric Particles

##### Carbon Nanotubes

Carbon nanotubes are carbon-based tubular structures 1 nm in diameter and 1–100 nm in length [40]. These structures can be obtained by wrapping a single layer of graphite called graphene into a seamless cylinder. The configuration of carbon nanotubes includes single-walled nanotubes (SWNTs), multi-walled nanotubes (MWNTs), and C60 fullerenes. The size and stable geometric shape of carbon nanotubes make them an attractive non-polymeric carrier for therapeutic agents. Particularly, SWNTs and C60 fullerenes have internal diameters of 1–2 nm, which is equivalent to about half of the average DNA helix diameter [41]. The SWNTs and MWNTs can enter the cell by endocytosis or by direct insertion through the cell membrane. Fullerenes differ in the arrangement of their graphite cylinders and the presence of a high number of conjugated double bonds in their core structure. Experiments with fullerenes have shown that they can be used for the delivery of therapeutics like antibiotics and antiviral and anti-cancer agents [42,43,44,45]. Additionally, they can protect the injured mitochondria by providing free radicals [46]. This feature allows for the tissue-selective targeting of mitochondria that can be used for delivering therapeutic agents [47].

##### Nanodiamonds (NDs)

Nanodiamonds (NDs) are members of carbon-based nanomaterials with a diameter of less than 100 nm and different shapes with two types of discrete facets, which are generated from various methods, such as the detonation, chemical vapor deposition (CVD), and high-pressure/high-temperature methods [48,49]. The oldest and most commonly used ND preparation is the detonation method, in which NDs are produced by activating a controlled explosion on carbon-containing precursors in a closed chamber. NDs that are prepared by this method usually contain sp^2^ carbon on the surface, and the electrostatic potential of surface is dependent on shape and structure of the NDs [50,51,52]. The CVD method is preferable for depositing NDs onto various substrates as thin films. The produced ND thin films are of high quality with low defects [53].

NDs have unique properties, including surface electrostatic properties, low cytotoxicity by a chemically inert core, and low photo-bleaching by the addition of nitrogen defects and can be functionalized by immobilization of various types of biomolecules, which make them remarkable for biomedical applications such as magnetic resonance imaging (MRI), synthesis of contact lenses, and drug delivery for cancer therapy. NDs can be coupled with gadolinium [Gd] (III) as a contrast agent for MRI, and the signal generated from this complex is several times higher compared to Gd (III)-based contrast agents [48,49,50].

##### Metallic Nanoparticles

Metallic nanoparticles used in medical applications are 1–100 nm in size and mostly made up of cobalt, nickel, iron, gold, and their respective oxides like magnetite, maghemite, cobalt ferrite, and chromium dioxide. They can be synthesized and modified with versatile functional chemical groups, which allows them to be decorated with various molecules including therapeutic agents, biological molecules like peptides, proteins, and DNA. As a carrier, they provide unique characteristics such as magnetic properties besides stability and biocompatibility. Thus, magnetic nanoparticles can be targeted to a specific location in the body by using an external magnetic field. Magnetic susceptibility, defined as the ratio of induced magnetization to the applied field, is an important parameter for their medical use. For example, super-paramagnetic iron oxide nanoparticles (SPIONs) have a large magnetic susceptibility, and thus, they are widely used in clinics as contrast agents for magnetic resonance imaging [54]. Likewise, super-paramagnetic properties facilitate the stable delivery of therapeutic agents to the body/cell and proper accumulation at the target tissue providing a reproducible and safe treatment approach [41,55]. When metallic nanoparticles are subjected to an alternating magnetic field, they can produce heat that is called magnetic hyperthermia, which enables their use in the ablation of tumors for cancer treatment [56,57].

Gold nanoparticles (AuNP) are widely used metallic nanoparticles, especially in cancer diagnosis and therapy. The reason for this is the unique optical and localized surface plasmon resonance (LSPR) and relatively low cytotoxicity due to the inert nature of gold. When the light with appropriate wavelength is administered to AuNPs as external stimuli, due to the LSPR property, they exhibit photothermal conversion and heat up the targeted tumor tissue to kill cancer cells. Besides, AuNPs are used for drug delivery, where (at the targeted site) the light irradiation can trigger the drug release [58,59]. Moreover, AuNPs’ optical and LSPR properties can be tailored for applications such as imaging, optical and electrochemical detection, diagnosis, and photothermal therapy [60,61].

##### Quantum Dots

Quantum dots (QDs) are tiny particles or nanocrystals of a semiconducting material with diameters in the range of 2-10 nm. These particles consist of a semiconductor inorganic core such as CdSe and an aqueous organic coated shell such as ZnS [62]. QDs produce distinctive fluorescence colors that are partly the result of unusually high surface-to-volume ratios for such particles. The core structure of QDs determines the color emitted, while the outer aqueous shell can be used for conjugation of biomolecules such as peptides, protein, or DNA [63]. QDs can also carry a cap, which improves their solubility in aqueous buffers. Due to their narrow emission, bright fluorescence, and high photo-stability, QDs can be used for tracking therapeutic agents within the cells/tissues [64,65]. Although the medical use of QDs is still debated, their surfaces for versatile bioconjugation, adaptable photophysical properties for multiplexed detection, and superior stability for longer investigation periods make them a superior candidate than other fluorescence agents.

##### Silica-Based Nanoparticles

Silica-based nanoparticles offer considerable advantages in nanotechnology due to their applicability for designing complex systems and cost-effectiveness. Their specific surface characteristics, porosity, and capacity for functionalization make them attractive tools for therapeutic delivery [66]. Silica nanoparticles have a large surface area covered with polar silanol groups, which are favorable for water adsorption and improve the stability of therapeutic agents. In addition, silica-based nanoparticles have ability to interact with nucleic acids, which allows their use as targeted delivery vehicles [67]. Their nanopore size and density can be tailored to achieve a constant delivery rate. Moreover, encapsulation of therapeutic agents within silica-based nanoparticles provides solid media for the delivery of agents. Pores of silica nanoparticles can be capped with various stimuli-responsive molecules to increase the rate of drug release in the targeted tissue. For instance, mesoporous silica nanoparticles capped with β-cyclodextrin were developed to release the encapsulated drug at the acidic tumor tissue [58]. Combination of these nanoparticles with contrast agents such as gold, silver, iron oxide, organic dyes, and quantum dots facilitates their tracking in biological systems [68]. Furthermore, these nanoparticles are used as additives in pharmaceutical production to improve the mechanical properties and the biocompatibility of the product.

#### 3.1.3. Lipid-Based Nanoparticles

##### Liposomes

Liposomes are vesicles synthesized through the hydration of dry phospholipids. They can be prepared in distinct structure, composition, size, and flexibility with a variety of lipid molecules and further surface modification. One of the most important advantages of liposomes is their ability to fuse with cell membrane and release their contents into the cytoplasm, which makes them suitable intelligent carrier systems for targeted delivery. The simplest liposome is composed of a lipid bilayer surrounding a hollow core with a diameter of 50–1000 nm. The therapeutic molecules can be loaded into this hollow core for delivery [69,70,71]. Depending on the number of bilayers, they are classified into three basic types: multilamellar, small unilamellar, and large unilamellar. Multilamellar vesicles consist of several lipid bilayers separated from one another by aqueous spaces. In contrast, unilamellar vesicles consist of a single bilayer surrounding the entrapped aqueous space. These structural properties allow them to carry both hydrophobic and hydrophilic molecules. Hydrophilic molecules can be carried in the aqueous interior of the liposome, while hydrophobic molecules can be dissolved in the lipid membrane [72]. Additionally, more than one type of drug can be loaded either within two compartments (lipid and aqueous) or several aqueous layers of multilamellar liposomes. This also allows different drug molecules to be released in sequence with dissociation of layers from the outer shell to the inner core [73]. The neutral or positively charged small liposomes have higher circulation time when compared to large, unmodified liposomes [74]. Moreover, surface modifications can be obtained by either coating it with a functionalized polymer or PEG chains that improve targeted delivery and increase their circulation time in biological systems [75]. The liposomes are investigated for a wide variety of therapeutic applications, such as cancer diagnostic and therapy, vaccines, brain-targeted drug delivery, and anti-microbial therapy [71].

##### Exosomes

Exosomes are naturally formed and secreted by various types of cells. They are endosome-derived extracellular vesicles with a size of 30–150 nm and usually exist in different body fluids such as saliva, blood, urine, and breast milk [76]. Exosomes are cell membrane-like lipid bilayer vesicles, which contain various substances, including RNA, DNA, glycolipids, and proteins [77,78]. Exosomes play an important role in intracellular communication by transferring various compounds in physiological mechanisms such as immune response, neural communication, antigen presentation in diseases like cancer, cardiovascular disease, diabetes, and inflammation [76,79]. Since these vesicles can be isolated from a patient’s bodily fluids, allogenic exosomes have an advantage over the immune system, which can easily protect the cargo from rapid clearance and improve the drug delivery to targeted sites [80]. Therefore, studies on the potential of exosomes for the use as drug delivery carriers for cancer and autoimmune diseases, diagnostic biomarkers of cancer, and even for tissue regeneration are emerging [81,82].

##### Solid Lipid Nanoparticles (SLN)

Solid lipid nanoparticles (SLN) are aqueous colloidal dispersions comprised of a lipid matrix that is solid at room and body temperatures. Surfactants improve their stability, whereas the choice of lipid affects the drug delivery characteristics. The size of SLNs varies from 10 to 1000 nm depending on the production approach [83]. SLNs as a sub-category of lipid carriers can encapsulate very high amounts of lipophilic drugs as well as hydrophilic drugs and nucleic acids, making them versatile drug delivery vehicles [84,85]. SLNs can be decorated or loaded with various moieties, including antibodies, magnetic nanoparticles, pH sensitive lipids/polymers to modulate targeted delivery, and stimuli-responsive drug release [86,87]. They are shown to be effective carriers for cancer, pulmonary, and oral drug delivery purposes [88,89].

### 3.2. Nanocrystalline Particles

Nanocrystalline particles, or nanocrystals, are carrier-free drug particles with a crystallite size of only a few nanometers. Nanocrystal formulations are widely prepared for poorly water-soluble drugs suffering from limited bioavailability and absorption as a highly cost-effective approach. Generally, the size reduction is a suitable way to enhance the bioavailability of agents, where the dissolution velocity is the rate-limiting step. The crystalline structure leads to an increased overall surface area and thus increases dissolution velocity. This characteristic improves the solubility, which is important especially when the therapeutic index of the agent is limited due to absorption problems. Relatively, nanocrystalline particles enable the quick absorption of therapeutic agents due to their fast dissolution, offering an advantage for agents that need to work fast. By modifying the nanocrystal surface, it is possible to achieve a prolonged or targeted release, allowing for the use of therapeutic agents in low doses and decreasing side effects [90].

## 4. Targeted Delivery Applications of Therapeutic Nanoparticles

Targeted delivery refers to the successful direction of therapeutic agent and its dominant accumulation within a desirable site. For the efficient targeted delivery, the agent-loaded system should be retained in the physiological system for the preferable time, evade the immunological system, target specific cell/tissue, and release the loaded therapeutic agent [91]. Currently, targeted delivery of nanoparticles is widely studied in cancer treatment. Over 20% of the therapeutic nanoparticles already in clinics or under clinical evaluation were developed for anti-cancer applications. In addition, related research has focused on nanoparticle-mediated therapy for some other diseases, such as neurodegenerative, infectious, autoimmune, etc. diseases. The subsequent section provides up-to-date applications of therapeutic nanoparticles as targeted delivery systems in several diseases, and Table 1 summarizes the Food and Drug Administration (FDA)- and the European Medicines Agency (EMA)-approved nano-drug formulations since 2009.

### 4.1. Cancer

Cancer is one of the major causes of death, and chemotherapy is widely used as a treatment approach for various cancer types. However, chemotherapeutic agents suffer from the lack of aqueous solubility, exhibit dose-dependent toxicity, and their tumor specificity is inadequate [108]. Multidrug resistance is another challenge in chemotherapy, which mainly occurs due to increased efflux pumps that are responsible for the export of anti-cancer agents from cell membrane [109].

Recent developments of nano-delivery systems overcome these limitations targeting directly the cancer cell, delivering the agent at a controlled rate, and optimizing the therapeutic efficacy [110]. A variety of nanoparticles has been developed for delivery of anti-cancer agents, and two major mechanisms are used to deliver them at the tumor site: Passive targeting and active targeting [111]. Passive targeting is based on the accumulation of therapeutic agent in the tumors due to their distinctive features compared to normal tissues. Tumors have leaky vasculature and defective lymphatic drainage promoting the delivery and retention of therapeutic nanoparticles, commonly referred to as the EPR effect [112]. Nonetheless, nanoparticles encounter several obstacles during passive targeting. Mucosal barriers or non-specific uptake of particles on the way to their target limit their efficiency. In contrast, active targeting achieves selective recognition of the targeted cells by carrying ligands at the surface of nanoparticles that bind to receptors or stimuli-based carriers [113,114].

Currently, the majority of FDA-approved therapeutic nanoparticles are developed as a re-formulation of chemotherapeutic drugs combinations with polymeric nanoparticles. The first nano-drug for cancer treatment was a PEGylated liposomal formulation of doxorubicin (Doxil^®^, Caelyx^®^). Doxil is formulated with sterically stabilized liposomes composed of phospholipids, cholesterol, and a lipopolymer (PEG) to achieve extended circulation time and eliminate RES. It is less than 120 nm and thus takes the advantage of EPR in accumulation in the tumor and in resulting in decreased cardiotoxicity [115]. Another example of liposomal drug formulation is Marqibo^®^, which is approved by the FDA for the treatment of acute lymphocytic leukemia in adults [92]. The liposomal formulation of Marqibo^®^ (Vincristine sulfate) is generated from sphingomyelin and cholesterol, which greatly improves the circulation time and expedite dose intensification as compared to standard Vincristine [116]. There are several lipid-based formulations under clinical trials as well [117,118]. For instance, one of the first line cancer chemotherapy agents, cisplatin, is formulated in soy phosphatidylcholine (SPC-3), cholesterol, dipalmitoyl phosphatidyl glycerol (DPPG), and methoxy-PEG-distearoyl phosphatidyl ethanolamine (mPEG2000-DSPE) under the brand name Lipoplatin^®^ (Regulon, Inc.). Lipoplatin^®^ pre-clinical trials revealed less nephrotoxicity in comparison to the conventional drug. Consequently, The European Medicines Agency (EMA) announced Lipoplatin^®^ as an orphan drug for the treatment of pancreatic adenocarcinoma, breast cancer, and advanced gastric cancer [119,120,121,122].

Drug conjugates are arranged to facilitate either the targeted delivery of cytotoxic drugs to tumor cells or decreased systemic cytotoxicity of the drug [123]. The FDA approved such an antibody-drug conjugate namely Kadcyla^®^ for the treatment of HER2+ breast cancer. The drug Kadcyla^®^ is formed by covalent conjugation of maytansine derivative DM1 to transtuzumab (known as Herceptin^®^) through the lysine residues of the antibody [124,125]. Thus, DM1 is delivered to HER2+ breast cancer cells through transtuzumab recognition of HER receptor, and internalized DM1 triggers apoptosis. The albumin-conjugated nanoparticle version of the anti-cancer drug paclitaxel (Abraxane^®^) or a re-formulation of the rapamycin drug with micellar nanoparticles (Rapamune^®^) are other FDA-approved therapeutic nanoparticles with lower side effects and improved therapeutic indices over their conventional counterparts [126]. Table 2 summarizes the therapeutic nanoparticles evaluated in the pre-clinical or clinical trials.

### 4.2. Infectious Diseases

The major therapeutic approach for infectious disease is the use of anti-microbial drugs. However, pathogens can become resistant, where anti-microbial drugs become therapeutically insufficient. In that case, administration dose and frequency are increased, which results in increased side effects and toxicity. Besides, many pathogens are located intracellularly in an active or latent state, which prevents the access of anti-microbial drugs [146,147]. The use of nano-delivery systems promises to overcome such limitations. Therefore, there is a growing interest to generate nanotechnology-based formulations against various pathogens, such as bacteria, viruses, fungi, or parasites.

Application of nano-delivery for the treatment of infectious diseases includes both polymer-based and non-polymeric nanoparticles, as well as liposomes that improve the anti-microbial activity of drugs [148]. Ciprofloxacin is a broad-spectrum antibiotic prescribed for lung infections. Lipoquin™, the liposome formulation of ciprofloxacin, is designed as an inhaled formula for sustained release of up to 24 h and thus eliminated the systemic effects of the high-dose antibiotic [149]. Similarly, the anti-fungal liposomal carrier Ambisome^®^ (amphotericin B) is designed to reduce associated toxicity of amphotericin B [150]. Due to its low systemic toxicity, liposomal amphotericin B can be used for highly immunocompromised patients with HIV infection or disseminated histoplasmosis [151].

Nanoparticles can also be fabricated into anti-viral drugs. Virosomal vaccines are the carriers of viral adjuvants such as viral glycoproteins, toll-like receptor, or protein fractions. Such vaccines, e.g., Inflexal^®^ V for hepatitis-A and Epaxal^®^ for influenza, are already used in clinics for therapeutic purposes [152]. Virosomal vaccine formulations are also being investigated, for instance in clinical trials for Ebola virus (EBOV). That approach creates safer viral vaccines and induces strong and long-lasting immune response.

Anti-microbial nano-drugs are also used to decorate medical devices to eliminate biofilm formation [153], such as AgNPs in central venous catheters [154]. Furthermore, there are some nanoparticles used in diagnosis or as medical devices like Verigene^®^, Silverline^®^, Acticoat™, or Endorem™ SPIONs [105,155,156].

A short list of therapeutic nanoparticles against resistant strains and some nano-delivery systems used for prevention and treatment against bacterial infection can be found in Table 3.

### 4.3. Autoimmune Diseases

Rheumatoid arthritis (RA) and acquired immunodeficiency syndrome (AIDS) are the main two diseases being treated using nano-delivery systems.

RA is one of the common and severe autoimmune diseases affecting almost 1% of the world’s population. Despite the cause being unknown, the complex interaction between immune mediators is likely responsible for the bone and cartilage destruction. New therapy approaches are able to improve the quality of the patient’s life; however, a restricted administration route and the requirement of repetitive long-term treatment result in systemic adverse effects [171]. Nanoparticle systems are promising for the delivery of therapeutic agents particularly to target inflamed tissue (synovial membrane), thereby preventing systemic and undesired effects. Certolizumab pegol (CZP) is a TNF-α inhibitor widely used in clinic [161,172]. Nano-formulation of CZP with PEG increases its half-life to ∼14 days, and clinical trials have shown promising results for the long-term treatment on RA patients [173]. Targeting inflamed tissues by using stand-alone C60 fullerenes (non-drug loaded) showed promising results in RA treatment by reducing synovitis and alleviated bone resorption and destruction [174].

Acquired immunodeficiency syndrome (AIDS) is another autoimmune disease lacking treatment. Current clinical therapy is called highly active anti-retroviral treatment (HAART), which consists of a combination of at least three anti-HIV medications suppressing human immunodeficiency virus (HIV) replication. Although this therapeutic approach has contributed to a decreased mortality rate, it is not fully effective [175]. Recently, nano-delivery systems are under development based on polymeric and liposomal nano-carriers to provide a target-specific and sustained release formulation of anti-HIV drugs. The goal is to improve efficiency of anti-HIV treatment and limit systemic side effects [176]. For instance, efavirenz is loaded into poly(propyleneimine) dendrimers (TuPPI), which are decorated with Tuftsin. Final TuPPI particles were able to recognize mononuclear phagocytic cells through Tuftsin and resulted in significantly higher uptake in HIV infected macrophages compared to uninfected cells [177]. Additional examples of nanoparticle drug formulations for AIDS therapy are summarized in Table 4.

### 4.4. Cardiovascular Diseases

Cardiovascular disease (CVD) affects the cardiovascular system, vascular systems of the brain and kidney, and peripheral arteries. Despite many novel therapeutic strategies such as gene delivery and cell transplantation, heart failure is still a leading cause of mortality worldwide [186]. Utilization of nanoparticle-based formulations to treat cardiovascular diseases is mostly focused on targeted delivery and increasing bioavailability for vascular restenosis.

As a nanoparticle drug for restenosis, liposomes formed by phosphatidylcholine and cholesterol were loaded with small drug sirolimus and coated with chitosan. The resulted liposomal sirolimus is shown to significantly inhibit vascular restenosis [187]. Another drug, carvedilol is a β-blocker widely used for the treatment of hypertension, myocardial infarction (MI), congestive heart failure, and post-MI left ventricular dysfunction. However, its low water solubility and extensive pre-systemic metabolism limit its bioavailability. The niosome-based nanoparticle formulation encapsulating carvedilol reached ~1.7–2.3-fold higher plasma concentrations compared to the free drug, resulting in enhanced bioavailability and improved therapeutic effect [188]. Similarly, resveratrol is a cardio-protective polyphenol with low bioavailability and water-solubility. Its nano-formulations both as solid lipid nanoparticle and liposome showed enhanced oral bioavailability and controlled release [189].

Angiogenic therapy of myocardial ischemia with vascular endothelial growth factor (VEGF) is a convenient approach to overcome hypoxia-dependent side effects. Polymeric particles loaded with VEGF have been proposed as a promising system to improve vasculogenesis and tissue remodeling in an acute myocardial ischemic model [190,191]. A targeting nano-delivery system in atherosclerosis is also achieved to visualize and treat atherosclerotic lesions by using magneto-fluorescent nanoparticles or ligand-binding polymeric micelles [192].

### 4.5. Neurodegenerative Diseases

Neurodegenerative diseases (NDs) are characterized via the progressive loss of the function of neurons, which subsequently causes neuronal death. Patients with NDs, such as Alzheimer’s disease (AD), Parkinson’s disease (PD), and multiple sclerosis (MS), have symptoms related to movement, memory, and dementia as a result of gradual loss of neurons. Although significant progress has been achieved in the treatment of NDs, the therapeutic strategies are limited due to the restrictive structure of the blood–brain barrier (BBB). The BBB is a highly selective semipermeable membrane, which separates the circulating blood from the brain and prevents the passage of the majority of the molecules in circulation so that central nervous system homeostasis is maintained [193]. Due to the highly selective nature of the BBB, only a small portion of therapeutic drugs can reach the brain. Therefore, high doses are required leading to adverse systemic effects. Nanoparticle-based therapeutic approaches in NDs mainly focus on targeted delivery and sustained local release of therapeutic agents into the affected area of the brain after crossing the BBB [194,195].

The aggregation of the amyloid-β (Aβ) peptide into amyloid plaques is the main pathological feature of AD, and current treatments include cholinesterase inhibitors (donepezil, rivastigmine, galantamine) and *N*-methyl-d-aspartate (NMDA) receptor antagonists (memantine) [196]. Reformulation of clinically used drugs with polymeric nanoparticles, non-polymeric quantum dots, and lipid-based nanoparticles enables their passage through BBB and reduces the side effects compared to free drug administration [197,198,199,200,201,202]. Concerning nano-delivery systems, there are also other attempts to cross the BBB and to reduce Aβ aggregates by using several neuroprotective compounds like metal chelators and various NMDA antagonists of anti-amyloids [203,204].

Parkinson’s disease (PD) is another type of neurodegenerative disease characterized by the selective degeneration of dopaminergic neurons and by the existence of α-synuclein as well as protein inclusions in neurons named Lewy bodies [205]. Dopamine replacement therapies are presently the most used strategy for PD treatment, since this class of drugs can help to improve the symptoms in motor neurons and is able to slow down the progression of the disease. However, the effect of these drugs on behavior and cognition is still in debate [206]. Recent research activities in nano-delivery focused on the development of therapeutic nanoparticles based on different strategies. Targeted delivery of dopamine using polymeric nanoparticles or liposomes is one of the nanoparticle-based therapeutic approaches in PD treatment [207]. Several studies use various drugs (Ropinirole, Bromocriptine, Mitoapocynin, Apomorphine) encapsulated with liposomes or polymeric nanoparticles in order to improve sustained release of drugs and to reduce undesired effects of conventional PD therapy [208,209,210]. Anti-inflammatory strategies are also developed by using polymeric nanoparticles or PEGylated liposomes to prevent neuronal cell death in PD [211,212,213]. As a neurotrophic strategy, PEGylated nanoparticles loaded with h-GDNF (glial cell-derived neurotrophic factor) improve locomotor activity and decrease the loss of dopaminergic neurons, which results in enhanced dopamine levels [214,215]. Moreover, polymer-based biodegradable nanoparticles have been engineered as cell therapeutics allowing stem cells to repair damaged nerves [216]. On the other hand, several groups proposed therapeutic nano-systems for the delivery of genetic material (DNA, RNA, and oligonucleotides) to inhibit faulty gene expression or synthesize therapeutic proteins in affected cells [217]. Although significant improvement in clinical symptoms is observed in advanced PD patients who underwent gene therapy, this approach is still a contradictive issue because of the heterogenic pathology of PD [218].

Despite many research articles on the development of novel therapeutic nanoparticles published for AD and PD, only few approaches have been reported for other neurodegenerative diseases, like amyotrophic lateral sclerosis (ALS) and multiple sclerosis (MS). ALS is a progressive neurodegenerative disease affecting motor neurons responsible for controlling voluntary muscle movements (chewing, walking, and talking) in the brain and spinal cord. Clinically, progressive muscle weakness results in death due to respiratory failure. To date, the only agent approved for treatment of ALS is Riluzole. Loading Riluzole in solid lipid nanoparticles promotes the delivery of the drug to the CNS (central nervous system), and pH-based controlled release is achieved with lower random biodistribution in organs such as the liver, spleen, heart, kidneys, and lung [219,220]. MS is characterized by the destruction of the protective coating (myelin sheath) on nerves of the central nervous system, which causes a faulty relay of instructions from the brain to the body. The conjugation of a glutamate receptor antagonist with a non-polymeric fullerene derivative nanoparticle is able to rescue the clinical progression of chronic MS in an in vivo model [221]. In 2015, Glatopa^®^ was approved by the FDA as the first generic drug, which is derived from the only therapeutic peptide, also approved by the FDA for the treatment of MS, glatiramer acetate (also known as the Copaxone^®^) [222]. Glatiramer acetate is a random copolymer and a synthetic peptide composed of l-lysine, l-alanine, l-glutamic acid, and l-tyrosine, which could suppress inflammatory responses by blocking MHC-II and changes the population of T-cell [102].

### 4.6. Ocular Diseases

Current ocular therapies taking advantage of nanomedicine can be enumerable as mydriatics or cycloplegics miotics, infection, and inflammatory, as well as diagnostic and surgical, adjuvants. However, several barriers, including the mucoaqueous tear layer, corneal epithelium, and the blood-retina barrier, make the eye impermeable for most therapeutic agents [223]. Targeted nano-delivery systems offer advantages in ocular disease therapy by lowering eye irritation and enhancing the bioavailability by providing a route of entry to the eye [224]. The most widely used nano-delivery systems are polymeric nanoparticles and liposomes developed for targeting the drugs to the right compartment of the eye, initially by increasing residence time on the tear film and enhancing corneal permeability [225,226]. Nano-formulation of the drug pranoprofen with the polymer PLGA (poly (lactic-co-glycolic acid) significantly enhanced its ophthalmic delivery and local anti-inflammatory and analgesic effects of the drug [227]. Similarly, chitosan-based polymeric nanoparticles encapsulating cefuroxime, diclofenac, or dexamethasone improved ocular bioavailability of the drugs [228]. These nanoparticles are able to interact with ocular surface while protecting the drug from metabolic degradation and extending pre-corneal residence [229]. Similarly, lipid-based nanoparticles loaded with brimonidine were used to treat an ophthalmic disease, glaucoma [230,231,232]. Immunologic graft rejection is a challenge in corneal transplantation. PLGA- or PEG-based polymeric formulations of dexamethasone and curcumin prevent the rejection of corneal graft due to the sustained release of the corticosteroids [233,234].

### 4.7. Pulmonary Diseases

Pulmonary lung diseases include asthma, chronic obstructive pulmonary disease (COPD), cystic fibrosis, pulmonary tuberculosis, and idiopathic pulmonary fibrosis (IPF) [235]. These diseases are often fatal, and there is no effective treatment for completely restoring lung function. Conventional therapeutics are applied either systemically or locally to the lungs in the form of inhalers. Inhalation of free drugs, where the active molecules are in aerosol form, can lead to burst release and thus high lung toxicity. Moreover, the size of the aerosol limits the efficiency of the drug, as molecules >5 μm cannot pass the upper respiratory tract, particles between 1–5 μm mostly settle at the lower respiratory tract, and particles <1 μm stay suspended in air and exhaled. Nanoparticle-based delivery systems enable enhanced bioavailability, controlled release, and decreased dosage and application frequency. For the development of nanomedicine inhalation formulations, natural polymeric nanoparticles such as gelatin, chitosan, and alginate, as well as synthetic polymers like poloxamer, PLGA, and PEG, are widely used [236,237]. Moreover, polyamidoamine (PAMAM) dendrimers assembled with anti-asthma beclometasone dipropionate (BDP) were effectively used for pulmonary inhalation [238]. Besides, lipid-, polysaccharide-, or polymer-based nanoparticles and metallic or carbon-based nanoparticles were utilized for vaccine delivery or pulmonary immune hemostasis [239].

### 4.8. Regenerative Therapy

Regenerative therapy focuses on the design and application of biocompatible materials, which can enhance the repair and regeneration of tissues by making use of their natural cellular mechanisms. Stem cell-based therapy is one strategy for promoting tissue’s natural repair or regeneration mechanism.

Over the years, there has been increased interest in the development and direct administration of therapeutic nanoparticles to promote bone regeneration [240]. The most commonly used nano-delivery systems for bone regeneration are synthetic (PLA or PLGA) or natural polymers (collagen, gelatin, albumin, and chitosan). Besides the polymeric ones, various formulations of non-polymeric nanoparticles (silica-based, metallic) have also been used as nano-delivery systems for bone regeneration. For example, calcium phosphate-based non-polymeric nanoparticles are mostly used due to their similarities to human bone [241,242,243]. Delivering several growth factors is one of the nanoparticle-based therapeutic strategies based on the stimulation of osteoblasts for bone formation [244,245,246,247]. Moreover, nano-delivery of synthetic molecules is used as another therapeutic strategy in bone tissue, which could suppress the bone-resorbing cells, the osteoclasts. The bisphosphonate drugs promote osteoclasts apoptosis and are thus widely used for osteoporosis treatment. Several types of polymeric or non-polymeric metallic nanoparticles have been used to deliver bisphosphonate drugs [248,249].

Implantations of bone-graft substitutes stimulating mineralization are an alternative and effective approach for bone and dental tissue regeneration. EquivaBone^®^ is a nanotherapeutic that consists of hydroxyapatite, carboxymethyl cellulose, and demineralized bone matrix. EquivaBone^®^ is used as an osteoinductive bone graft substitute and was approved by FDA in 2009 [250].

Another strategy for the use of therapeutic nanoparticles in bone tissue is reducing inflammation, particularly in the case of large wounds. Synthetic or natural polymeric nanoparticles loaded with anti-inflammatory agents are delivered into the infected area, which could inhibit both inflammation and osteoblast resorption [251,252].

## 5. Limitations and Disadvantages of Therapeutic Nanoparticles

Utilization of nanoparticles provides promising results for the treatment of a large variety of diseases from cancer to glaucoma. Nanomedical approaches based on nanoparticle technologies, unfortunately, come with some limitations and disadvantages. Nanoparticle toxicity, eluding from the phagocytic system, refraining from the physiological barrier, and generating immune response are only some of the issues that should be taken into consideration carefully while using them in living organisms [253].

In vitro and in vivo studies showed that there is a relation between the size and toxicity of therapeutic nanoparticles. As nanoparticle sizes get smaller, dispersion to the nucleus steadily increases, which in return can cause intrinsic toxicity both at cellular and systemic level [254,255]. Another obstacle with smaller nanoparticles is their aggregation tendency. For instance, smaller size of micelles, dendrimers, and QDs are inclined to aggregation that results in poor biodistribution [256,257,258,259]. Although surface functionalization with PEG is a very effective method for reducing accumulation in non-target organs, these nanoparticles are often referred to as “stealth” nanoparticles because they can elude from the phagocytic system and may provoke cellular toxicity [260].

Therapeutic nanoparticles combined with drugs currently used in medical applications provide various features to the drug and increase the efficiency of the treatment. However, Manzoor et al. found that drug concentration and penetration into the tumor areas might be limited with nanoparticle-based drug delivery systems due to the heterogeneities of vascular permeability. In order to overcome the problem, they suggested a controlled delivery by using drug-loaded liposomes that are being triggered by local heat for drug release in vitro [261].

QDs are another type of contradictive nanoparticles for medical applications. As mentioned above, QDs have characteristic properties for fluorescent emission; thus, they are widely used for imaging applications. Beside their benefits during diagnosis of the disease, QDs have non-negligible disadvantages such as high intrinsic cytotoxicity. Research related to cadmium-based quantum dots showed that there is leakage of metal ions in QDs, resulting in high toxicity in hepatocyte cultures [262,263,264]. Shao et al. showed that effects of QDs could differ depending on the coating types as liposome or polymer. Another research indicated that quantum dot-lipid complex (QD-LC) has little effect on normal human hepatic cells and selectively kills cancer cells in a dose- and time-dependent manner in vivo. Furthermore, QD-LC nanoparticles trigger reactive oxygen species (ROS)-mediated apoptotic c-Jun N-terminal kinase (JNK) pathway in human liver cancer cells [265].

## 6. Conclusions

During the last decade, development of nanoparticle-based therapeutic agents has been extensively studied, and nano-delivery systems are the area of prime importance for specifically targeting the desired area in the treatment of many diseases.

Currently, the majority of nanoparticles used for the targeting delivery approach are made of polymers or lipids. Even though polymeric nanoparticles demonstrate great advantages in disease therapy, they also present disadvantages such as difficulties in scaling up, usage of organic solvents in their fabrication process, biocompatibility, cytotoxicity, and immunogenicity. On the other hand, lipid-based nanoparticles exhibit the ability to cross hard-to-reach sites, even without any surface functionalization, because of their similarity to cell membrane. Thus, lipid-based nano-delivery systems are considered as the next generation of therapeutics.

As of today, therapeutic nanoparticles are mostly developed for the treatment or prevention of only one disease. However, researchers started to combine various drug molecules as well as various types of nanoparticles, thereby, the future of therapeutic nanoparticles is guided to the direction of multi-therapeutic nanoparticles to be designed for the treatment of more than one disease.

Although nanoparticle-based delivery systems contribute significantly to the targeted therapy with improved efficiency, reduced side effects, and better bioavailability, we still know very little about the metabolism, clearance, and toxicity of nanoparticles. To date, most of the published studies demonstrate the encapsulation of clinical drugs with nanoparticles. However, the studies on other therapeutics, like genes, enzymes, or DNA/RNA, are still limited. The significance of published data is often approved with patents; however, a few of them can pass the trials and become commercially available in the market. This suggests that additional studies are required about the formulation, fabrication, and toxicity of nanoparticles. Moreover, cost of nanomedicine and manufacturing at larger scale is another important issue needing to be addressed. The lack of financial input or the poor cost–benefit balance prevents the progress of therapeutics from their inception to the market. Consequently, understanding the characteristics of nanoparticles and their interactions with their biological environment, like their targeting receptors or mechanisms of action in disease pathophysiology, will enable us to overcome limitations and to establish novel strategies for the treatment, prevention, and diagnosis in many diseases, particularly untreatable ones.

Nanomedicine will be the future of medicine, and nanoparticle-based therapeutics lies at the heart of it. However, a long ground should be gained before prosperity. Most importantly, long-term safety/toxicity of the nanoparticles should be investigated. Meanwhile, the discoveries on disease mechanisms and new drugs will lead to ways of placing more efficient and safer nanoparticle-based therapeutics in treatment regimens.

## Figures and Tables

**Table 1:** Food and Drug Administration (FDA)- and European Medicines Agency (EMA)-approved therapeutic nanoparticles since 2009.

Nanostructure	Product	Nanoparticle Formulation	Drug	Indication(s)	Approval	Ref.
Liposomes	Marqibo^®^	Sphingomyelin and cholesterol	Vincristine sulfate	Acute lymphoid leukemia	FDA 2012	[92]
	Mepact^®^	1-Palmitoyl-2-oleoyl-sn-glycero-3-phosphocholine and 1,2-Dioleoyl-sn-glycero-3-phospho-l-serine liposomes	Mifamurtide	Non-metastasizing osteosarcoma	Europe 2009	[93]
	Onivyde^®^	Nanoliposomes	Irinotecan	Pancreatic cancer, Colorectal cancer	FDA 2015 Europe 2016	[94]
	Vyxeos^®^	Distearoylphosphatidylcholine, Distearoylphosphatidylglycerol, Cholesterol	Daunorubicin Cytarabine	Acute myeloid leukemia	FDA 2017	[95]
Lipid-based (Non-liposoma)	Onpattro^®^	Lipid nanoparticles	Transthyretin targeted siRNA	Transthyretin-mediated amyloidosis	FDA 2018	[96]
Polymer-based	Glatopa^®^	l-glutamic acid polymer with l-alanine, l-lysine, and l-tyrosine (Glatiramer)	-	Multiple sclerosis	FDA 2015	[97]
Protein-drug conjugates	Kadcyla^®^	Maytansine derivative, DM1	Trastuzumab	HER2+ breast cancer	FDA 2013	[98]
	Abraxane^®^	Albumin	Paclitaxel	Non-small lung cancer, Pancreatic cancer	FDA 2012 Europe 2005, FDA 2013 Europe 2008	[99]
	Krystexxa^®^	PEGylated uricase	Pegloticase	Gout disease	FDA 2010 Europe 2013	[100]
	Plegridy^®^	PEGylated interferon β-1a	Interferon β-1a	Multiple sclerosis	FDA 2014 Europe 2014	[100]
	Adynovate^®^	PEGylated factor VIII	Factor VIII	Hemophilia	FDA 2015	[101]
	Rebinyn^®^	Glycopegylated coagulation factor IX	Factor IX	Hemophilia	FDA 2017	[102]
Metallic nanoparticles	Feraheme^®^	Superparamagnetic iron oxide nanoparticle (SPION) covered with dextran	-	Anemia in chronic kidney disease	FDA 2009 Europe 2012	[103]
	Ferinject^®^	Nanoparticles of ferric oxide core-carboxymaltose shell	-	Iron deficiency anemia in chronic kidney disease	FDA 2013	[104]
	NanoTherm^®^	Nanoparticles of superparamagnetic iron oxide coated with amino silane	-	Glioblastoma, prostate, pancreatic cancer	Europe 2009	[105]
Nanocrystals	EquivaBone^®^	Hydroxyapatite	-	Osteoinductive bone graft substitute	FDA 2009	[106]
	Invega^®^ Sustenna^®^	Paliperidone palmitate	Paliperidone	Schizophrenia	FDA 2009/2015 Europe 2011	[107]
	Ryanodex^®^	Dantrolene sodium	Dantrolene	Malignant hyperthermia	FDA 2014	[100]

**Table 2:** Therapeutic nanoparticles under pre-clinical or clinical evaluation.

Nanostructure	Nanoparticle	Conjugated Drug	Evaluation	Ref
Dendrimer	Polyethylene glycol (PEG)-platinum	α-cyclodextrin	pre-clinical	[127]
	Polyamidoamine dendrimer	*N*-acetyl-cysteine	clinical/phase I	[128]
Micelle	Polypropylene sulfide-PEG- serine-folic acid zinc phtalocyanine	doxorubicin	pre-clinical	[129]
	PEG-polyaspartate polymeric micelle	paclitaxel	clinical/phase III	[130]
Carbon nanotube	PEGylated single walled CNT	epidermal growth factor (EGF), cisplatin	pre-clinical	[131]
Metallic nanoparticles	Hollow mesoporous copper sulfide nanoparticle with iron oxides	doxorubicin	pre-clinical	[132]
	Hollow mesoporous copper sulfide nanoparticle with hyaluronic acid	doxorubicin	pre-clinical	[133]
	PEGylated MoS nanosheets		pre-clinical	[134]
	Azo-functionalized magnetite nanoparticles	doxorubicin	pre-clinical	[135]
	PEGylated gold nanorods (AuNR)	doxorubicin	pre-clinical	[136]
	Iron oxide magnetic silica-gold nanoparticles		clinical/phase I	[137]
	PEGylated gold nanorod	aptamer	pre-clinical	[138]
Silica based nanoparticles	Peptide-functionalized mesoporous silica	lactobionic acid, doxorubicin	pre-clinical	[139]
	Transferrin mesoporous silica	doxorubicin	pre-clinical	[140]
	PEGylated mesoporous silica	amino-β-cyclodextrin, doxorubicin	pre-clinical	[141]
	mesoporous silica	cytochrome C conjugated lactobionic acid-doxorubicin	pre-clinical	[142]
	Hydroxyapatite nano-crystalline nano-structured silica gel		clinical/phase 0	[143]
Nanodiamonds	PEGylated nanodiamonds	doxorubicin	pre-clinical	[144]
	PEGylated nanodiamonds	irinotecan, curcumin	pre-clinical	[145]

**Table 3:** Therapeutic nanoparticles and nano-delivery systems for the prevention and treatment of bacterial infections.

Pathogen	Nanoparticle	Conjugated Drug	Evaluation	Ref
*C. Albicans*	Metallic nanoparticle (AgNP)	Fluconazole	In vitro	[157]
*E. Coli*	Metallic nanoparticle (AuNP and AgNP)	Ampicillin	In vitro	[158]
*E. Coli*	Metallic nanoparticle (ZnO-PEI)	Tetracycline	In vitro	[159]
Enterococci	Metallic nanoparticle (AuNP)	Vancomycin	In vitro	[160]
	Liposome		In vitro	[161]
HIV-infected cells	Polymeric nanoparticle (Micelle)	Nelfinavir, saquinavir	In vitro	[162]
*P. Aeruginosa*	Liposome	Polymyxin B	In vitro	[163]
*P. Aeruginosa*	Metallic nanoparticle (AuNP)	Ampicillin	In vitro	[158]
*Plasmodium* sp.	Liposome	Chloroquine	In vitro	[164]
*S. Aureus*	Chitosan NP	Vancomycin	In vitro	[165]
	Metallic nanoparticle (AuNP)		In vitro	[166]
	Polymeric nanoparticle (PLA NP)	Penicillin	In vitro	[167]
	Silica nanoparticle		In vitro	[168]
	Chitosan NP	Streptomycin	In vitro	[169]
	Liposome	β-Lactam, penicillin	In vitro	[170]
	Metallic nanoparticle (AuNP and AgNP)	Ampicillin	In vitro	[158,166]

**Table 4:** Therapeutic nanoparticle drug formulations for the treatment of AIDS disease.

Nanostructure	Nanoparticle	Conjugated Drug	Evaluation	Ref
Polymeric nanoparticle	Poly(hexylcyanoacrylate) nanoparticles	Zidovudine	Pre-clinical	[178]
	Poly(isohexyl cyanate) nanoparticles	Zidovudine	Pre-clinical	[179]
	Poly(propyleneimine) dendrimers	Efavirenz	In vitro	[177]
	PPI dendrimer	Efavirenz	In vitro	[180]
	PLGA nanoparticles	Ritonavir, Lopinavir, Efavirenz	Pre-clinical	[181,182]
	PBCA and MMA-SPM nanoparticles	Stavudine, Zidovudine, Lamivudine	In vitro	[183]
	Poly(epsilon-caprolactone)	Saquinavir	In vitro	[184]
Liposome	Mannosylated and galactosylated liposomes	Stavudine	In vitro	[185]
